# The Clock Is Ticking: Countdown to Metastases

**DOI:** 10.1371/journal.pgen.1006299

**Published:** 2016-09-22

**Authors:** Linda D. Siracusa, Karen M. Bussard

**Affiliations:** 1 Department of Microbiology and Immunology, Sidney Kimmel Cancer Center, Thomas Jefferson University, Philadelphia, Pennsylvania, United States of America; 2 Department of Cancer Biology, Sidney Kimmel Cancer Center, Thomas Jefferson University, Philadelphia, Pennsylvania, United States of America; Stanford University School of Medicine, UNITED STATES

Metastases cause more than 90% of the morbidity and mortality associated with human cancers. Gene expression signatures associated with cancer progression and metastasis serve as unique tools to assist in patient diagnosis, prognosis, and treatment. Various types of signatures have been identified, ranging from those that are tumor-intrinsic or specific to a particular cancer subtype [1] to genes associated with a specific clinical outcome (e.g., “poor prognosis gene signature”) [2], as well as to genes associated with the development of metastatic lesions [3]. Of those genes that drive cancer processes, some may function by acting on the primary tumor—causing rise of metastatic lesions—or at the metastatic site to promote colonization, survival, and incorporation of surrounding stroma. Identification of metastasis susceptibility genes is thus key for prediction of cancer risk and metastatic relapse.

## A Mouse Model for Breast Cancer Tumorigenesis

The process of metastasis is complex, and debate has ensued concerning the role of host gene variation in contributing to metastatic potential. Although evaluation and sequencing of human tumors is revealing insights, much work still needs to be done to achieve the goal of personalized medicine, in which sequence variants dictate a patient’s course of treatment. To understand how genetic background influences primary cancer development and subsequent metastases, Hunter and colleagues chose an elegant model system [4]—namely, a transgenic mouse in which the polyoma virus middle T antigen is under the control of the mouse mammary tumor virus (MMTV-PyMT) promoter on the FVB/NJ inbred strain background. MMTV-PyMT mice develop mammary cancers that metastasize to the lung [5].

**Fig 1:**
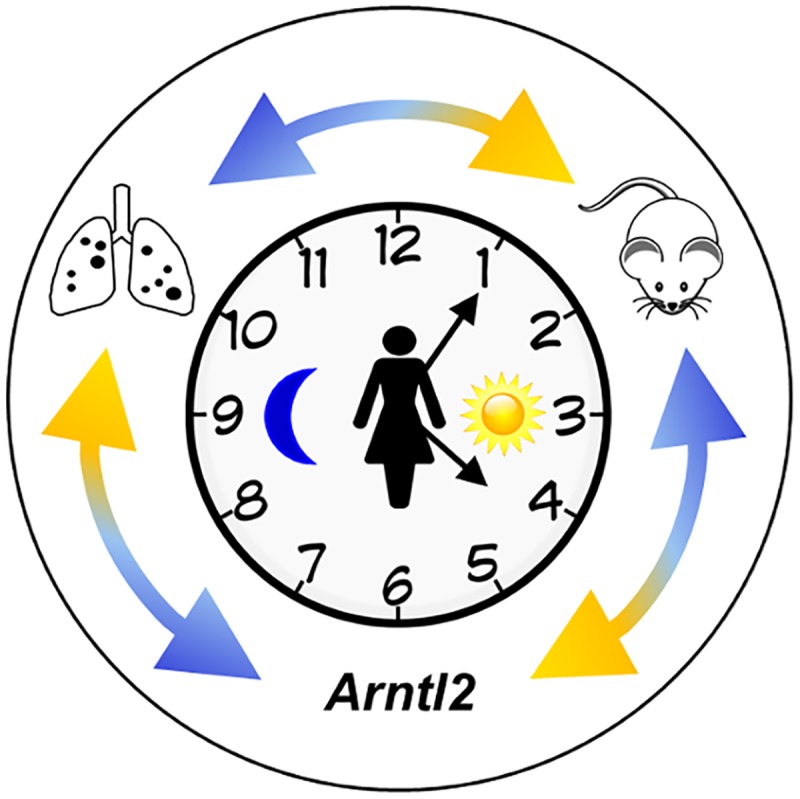
Identification of the *Arntl2* gene and its relationship to cancer metastases. The clock represents circadian rhythms. The external double arrows indicate how genetic studies using mouse models inform studies of human cancer, leading to the discovery of polymorphisms in the *Arntl2* gene that influence mammary cancer metastases to the lungs [7]. Black dots on the lung diagram represent metastatic lesions.

Using the MMTV-PyMT mice, Hunter and colleagues screened inbred strains to identify those that were susceptible or resistant to mammary tumor growth and metastases. By comparing the PAM50 gene signatures of primary mammary tumors, they demonstrated that tumor subtype is significantly impacted by the host genome [6]. Crossing MMTV-PyMT males with females from the genetically divergent MOLF/EiJ inbred strain, followed by crossing hybrid MMTV-PyMT males to FVB/NJ females, predisposed MMTV-PyMT N2 offspring to mammary tumors with gene signatures resembling estrogen receptor negative (ER-) breast cancers, thus tilting the model towards an aggressive form of breast cancer [6]. In this issue, the authors use quantitative trait loci (QTL) analyses to identify a region on mouse chromosome 6 that contains candidate genes for lung metastasis, including the Aryl hydrocarbon receptor nuclear translocator (*Arntl2*) gene (a member of the circadian clock; the *Arntl2* gene has also been called *Bmal2*, *Mop9*, and *bHLHe6*). Using an *Arntl2* knockout mouse, the authors demonstrate that absence of *Arntl2* increases the number of lung metastases but not metastatic latency or burden. Furthermore, the authors use CRISPR/Cas9 gene editing to recapitulate specific FVB/NJ polymorphisms in the MOLF/EiJ genetic background and show that sequence variants in the promoter region of *Arntl2* alter transcript levels, leading to changes in the metastatic potential of primary mammary tumors ([7]; this issue). Finally, they translate these findings to human cancers by evaluating sequence variants affecting *ARNTL2* expression and their impact on disease-free survival. This study demonstrates the power of mammalian model systems coupled with unbiased screens to identify understudied genes, test hypotheses using gene editing technology, and decipher mechanisms involved in metastatic spread (Fig 1).

## The *Arntl2* Gene Acts in a Tumor Cell–Autonomous Manner

The discovery of *Arntl2* was based on its sequence similarity to the *Arntl* and *Drosophila* Cycle (*cyc*) genes; *Arntl2* arose following duplication and divergence of the *Arntl* gene in vertebrates (the *Arntl* gene has also been called *Bmal1*, *Mop3*, and *bHLHe5*) and is linked to modulation of circadian rhythms [8]. Interestingly, Ha et al. established that alteration of *Arntl2* solely impacts cells of the tumor, with no impact on supporting stromal cells [7]; when hybrid *Arntl2* knockout mice were compared with wildtype *Arntl2* mice after orthotopic injection of syngeneic 4T1 mammary cells, there were no differences in the number of lung metastases, suggesting that *Arntl2* acts in a tumor cell–autonomous manner. Similar studies by Hunter and colleagues indicate that these properties are gene-specific, with other metastasis susceptibility genes (specifically *Cadm1*) impacting signaling and subsequent tumor progression in CD8+ T lymphocytes [9].

Most recently, Brady et al. showed a supportive role for *Arntl2* in driving lung adenocarcinoma and subsequent metastatic outgrowths [10]. *ARNTL2* expression was also shown to be up-regulated in colorectal cancers and correlated with tumor invasiveness [11]. Importantly, the relationship of *ARNTL2* to lung metastases may not be limited to breast cancer, as its paralog, the *ARNTL* gene, has been implicated in colorectal, hepatic, and other cancers [12,13].

## Specificity of Metastasis Susceptibility Genes to Tumor Subtype

It is now accepted that a family history of cancer correlates with increased risk and potential development of metastatic lesions. With the discovery of high-risk penetrant mutations in the *BRCA1* and *BRCA2* genes, it became evident that some breast cancers are inherited diseases [14,15]. Hunter and colleagues previously used the MMTV-PyMT mouse model to identify genes that influence metastatic progression [9,16]; however, these prior studies identified genes influencing metastasis of ER+ breast tumors, such as the *RRP1B* and *SIPA1* genes [17]. Their current study illustrates a link between metastasis susceptibility genes and breast cancer subtypes.

## Future Prospects

The current challenge is to identify genes and/or sequence variants predisposing individuals to cancer metastases. While in vitro assays allow for the investigation of proliferation, motility, and invasion, and in vivo models illustrate specific portions of the metastatic cascade, both systems do not fully recapitulate human cancer progression and the metastatic microenvironment. The vast genetic diversity present in inbred mouse strains, coupled with the ability to produce novel genetically engineered alleles with surgical precision, provide for mammalian model systems that closely mimic human cancer subtypes and enable identification of genes associated with inherited metastasis susceptibility. The report by Ha et al. describes a clever mating scheme that converted a predominantly ER+, luminal phenotype into a system that more closely resembles ER- tumors. Similarly, Lee et al. recently utilized QTL analysis of (TRAMP x PWK/PhJ)F2 mice to identify two novel candidate genes for prostate cancer metastasis susceptibility [18]. These types of studies illustrate the importance of designing creative model systems capable of recapitulating early stages of cancer development through progression and metastases to answer provocative questions pertaining to human disease.

Data collected from mammalian models, in concert with data generated by profiling human tissues, will facilitate the development of algorithms useful for predicting an individual’s specific risk of cancer and/or metastatic relapse, given their personalized gene signature. More importantly, the interchange of information between these systems may help solve important questions such as:

- Are there specific genes that govern whether and how long disseminated tumor cells of a given cancer type remain in a quiescent state?
- Are there specific metastatic genes that cause dormant cancer cell reawakening?
- Of the cancers that preferentially metastasize to the same organ, are there common genes causing directional metastasis?

Mammalian model systems are perfectly poised to test these and other questions, including ones regarding the emerging associations between dysregulation of clock genes and cancer (reviewed in [19]). Further intriguing issues revolve around the timing of cancer treatments, which may be adjusted to each patient’s circadian rhythms to result in better outcomes (reviewed in [20]). The answers will enhance our understanding of metastases as well as provide for better clinical management and development of preventive strategies.
